# MicroRNA profile changes in human immunodeficiency virus type 1 (HIV-1) seropositive individuals

**DOI:** 10.1186/1742-4690-5-118

**Published:** 2008-12-29

**Authors:** Laurent Houzet, Man Lung Yeung, Valery de Lame, Dhara Desai, Stephen M Smith, Kuan-Teh Jeang

**Affiliations:** 1 Molecular Virology Section, Laboratory of Molecular Microbiology National Institute of Allergy and Infectious Diseases, National Institutes of Health, Bethesda, Maryland 20892, USA; 2 Department of Infectious Diseases, Saint Michael's Medical Center, Newark, New Jersey, 07102, USA

## Abstract

MicroRNAs (miRNAs) play diverse roles in regulating cellular and developmental functions. We have profiled the miRNA expression in peripheral blood mononuclear cells from 36 HIV-1 seropositive individuals and 12 normal controls. The HIV-1-positive individuals were categorized operationally into four classes based on their CD4+ T-cell counts and their viral loads. We report that specific miRNA signatures can be observed for each of the four classes.

## Background

MiRNAs are single-stranded small oligoribonucleotides of 19–25 nt in size that originate from larger RNA polymerase II (RNAP II) transcripts [1-3]. They have been described in plants, invertebrates, and vertebrates. There is evidence that miRNAs function in cellular development, differentiation, proliferation, apoptosis, and metabolism [1,4,5]. Perturbed expression of miRNAs is also implicated in cancers and viral infections [6-11].

The course of human immunodeficiency virus (HIV-1) infection in cells is impacted by the action of several hundred host proteins [12-16]. Viral replication appears to be modulated also by the expression of human microRNAs [17-20]. In turn, the expression of HIV-1 proteins in cells [21] or the *in vivo *infection by virus [22] (as monitored by cells harvested from infected individuals) can change human miRNA profiles. To date, a systematic investigation of how human miRNA patterns vary at various stages of HIV-1 infection has not been performed. Here, using patient peripheral blood mononuclear cells (PBMCs), we present miRNA profiling of four classes of HIV-1 seropositive individuals. We report that HIV-1 infection generally resulted in the down regulation of most human miRNAs *in vivo*.

## Results

### MicroRNA expression is deregulated in HIV infected patients

**Figure 1:**
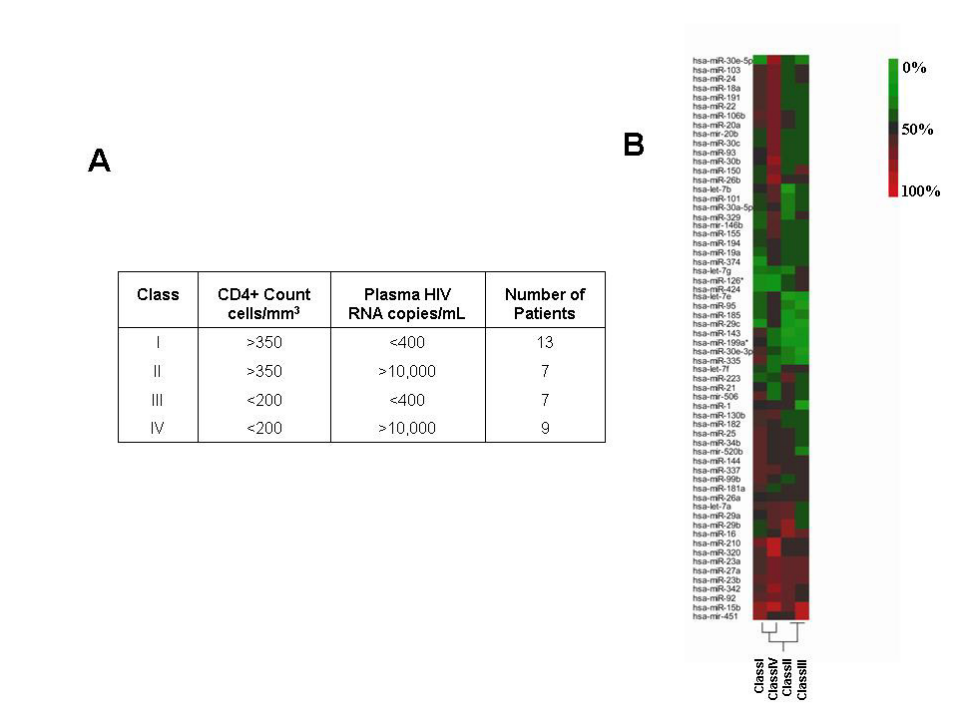
**Description of the four classes of HIV seropositive individuals and frequent miRNA changes in these individuals**. A) CD4+ cell counts and viral load classifications for the four classes of patients. B) Frequency heatmaps of the 62 most commonly changed miRNAs in the four classes of patients. 0% indicates that the enumerated miRNA is unchanged in any of the individuals in that class, while 100% means that all individuals in the indicated class are changed for that miRNA. "Change" is defined by at least a 2 fold down- or up- regulation when compared to normal control PBMCs. The color-key for the % frequency scale is at the top right.

Five PBMC cohorts were assayed in this study. The groups included normal anonymous blood bank donors, and anonymously labeled patient samples from four classes of HIV-1 seropositive individuals [i.e. patients with high CD4+ T cell count and low viral load (class I), high CD4+ T cell count and high viral load (class II), low CD4+ T cell count and low viral load (class III), and low CD4+ T cell count and high viral load (class IV) (Figure 1A)]. These four classifications of HIV-1 individuals are operationally defined; other ways to stratify patients are possible and merit additional consideration. Nevertheless, small RNAs were extracted from the phlebotomized PBMC samples, and the expression of 327 well-characterized human cellular miRNAs was analyzed using miRNA microarrays as previously described [21]. *En toto*, 12 normal and 36 discrete patient PBMCs were characterized by microarray miRNA profiling.

Primary PBMC samples are expected to show some degree of individual-to-individual variability. To analyze the raw miRNA readouts, we applied two levels of filtering. First, we considered only those miRNAs that were at least two fold or more changed (either up or down) when compared to normal controls. Second, we discarded miRNA changes that were not replicated in more than 50% of the patients in any of the four different classes. When these two filters were applied to the 327 miRNA readouts, 62 miRNAs satisfied both criteria (Figure 1B). The frequencies of these 62 miRNA changes were then compared between class I, II, III, and IV patients using JMP software and BRB array tools (see Materials and Methods). The resulting *in silico *clustering patterns indicated a closer relatedness in the frequencies of miRNA changes between class II and class III patients; and between class I and class IV patients (Figure 1B). It is unclear at this juncture what these relationships mean biologically.

### Class-specific signatures in HIV-1 patient PBMCs

**Figure 2:**
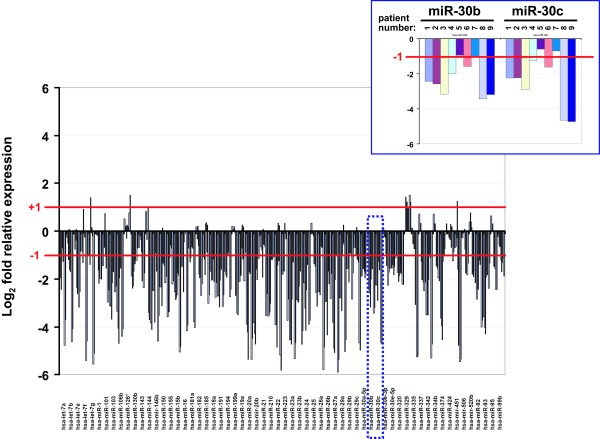
**A graphic representation of the indicated 62 miRNA readouts for the 9 class IV individuals**. Each vertical line represents a single miRNA value of relative expression [Log_2 _(class IV/normal PBMC)]. For each miRNA, there are 9 vertical lines corresponding to the 9 patients in class IV. Patient-to-patient variabilities are shown by the amplitude of the vertical lines as well as by occasional upticks for a miRNA when the majority of the values for that miRNAs were represented by downticks. +1 or -1 in the Y-axis represents the two-fold up- or down- cutoffs. Note that most values are downticks that exceed the -1 two-fold cutoff. The example inset at the upper right shows an enlarged view of the data set included in the dotted blue box.

Of the 62 frequently-changed miRNAs in the four classes of patients, 59 were down regulated while 3 were up regulated when compared to normal PBMCs (Figure 2). As expected, some polycistronic miRNA clusters such as miR-451 and miR-144; and miR-23a, miR-27a, and miR-24 were down regulated simultaneously. In figure 2, we show an example of the typical data points graphed from the microarray results for the 62 miRNAs from the 9 class IV patients. Similar patterns of mostly down regulated miRNAs were also observed for the other three patient classes (data not shown).

**Figure 3:**
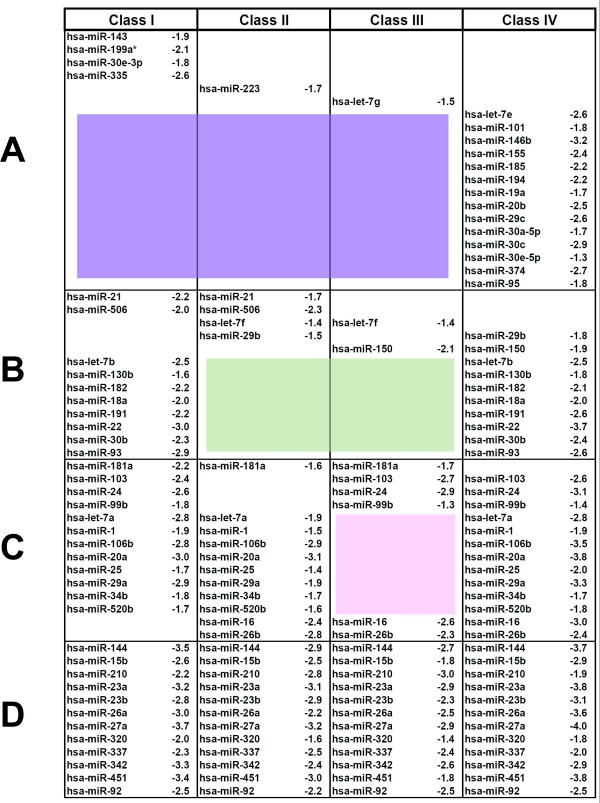
**Class-specific miRNA signatures in HIV infected individuals**. The 59 down regulated miRNA are tabulated based on their frequency in one (A), two (B), three (C) or all the four (D) classes of patients. The average fold down regulation is indicated for each miRNA by Log_2 _value. The colored areas highlight the absence of selected miRNAs in the corresponding class(es).

Since the vast majority of miRNAs were down regulated, we next asked whether these 59 miRNAs segregated into specific patterns (Figure 3). In parsing the results, we noted certain "signatures". For example, the down regulation of 14 mRNAs was specific to class IV, but was absent from class I, II or III; and the changes in 4 other miRNAs (hsa-miR-143, hsa-miR-199a, hsa-miR30e-3p, hsa-miR335) were unique to class I, but not observed in class II, III, or IV (Figure 3A). 8 other miRNAs were changed in both class I and IV patients, but not in class II or III patients (Figure 3B); while a further 8 miRNAs (hsa-let-7a, hsa-miR-1, hsa-miR-106b, hsa-miR-20a, hsa-miR-25, hsa-miR-29a, hsa-34b, and hsa-miR-520b) were changed in class I, II, and IV patients, but were absent from class III patients (Figure 3C). Lastly, 12 miRNA changes were present in all four classes of patients (Figure 3D). These patterns suggest class-specific "signatures" that plausibly correlate stage-specific miRNA alterations with the *in vivo *course of HIV-1 infection.

### miRNA profiles are changed in PBMCs treated ex vivo with T-cell activating or inactivating stimuli

**Figure 4:**
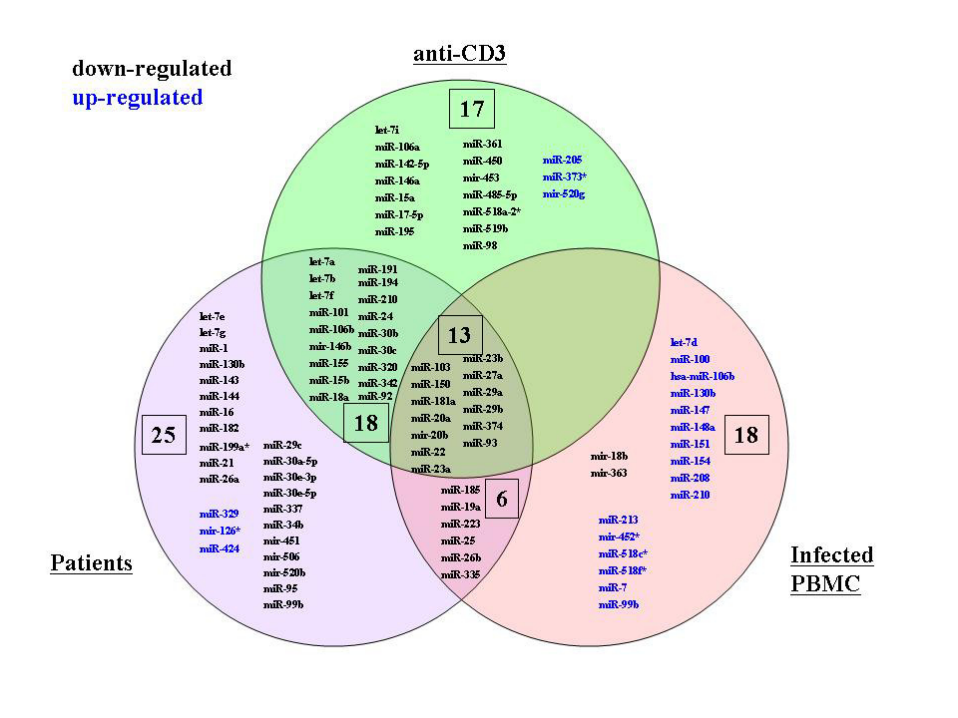
**Venn diagram of the overlap of miRNA profiles in patients, in stimulated PBMCs, and in virus infected PBMCs**. The miRNAs differentially expressed in patients, in anti-CD3 treated PBMCs, and in HIV-1 infected PBMC are depicted in three overlapping circles. The numbers indicate the miRNA counts in the indicated area.

In seropositive individuals, HIV-1 infects only a very small fraction of the circulating CD4+ T – cells. Thus, most of the PBMCs from our 36 patients (Figure 1A) are not infected by virus. The observed miRNA changes are likely indirect bystander results from systemic changes in activation status or cytokine levels in the infected individuals. To ask how the changes in patient miRNAs correlate with those seen from direct viral infection, we compared our 62 frequently changed miRNAs to those observed from cultured primary PBMCs that were infected *ex vivo *with HIV-1 pNL4-3. While there was some overlap between "Patients" miRNAs and "Infected PBMC" miRNAs, 50% or greater of the miRNAs in the two sets were discordant (Figure 4), indicating that a significant portion of the "Patients" changes could not be accounted by direct viral infection.

**Figure 5:**
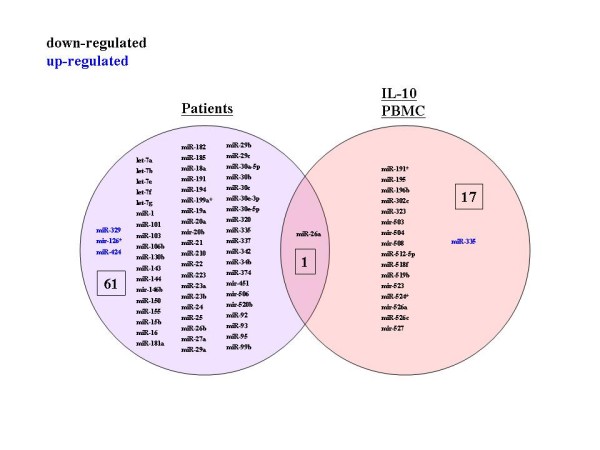
**Divergence between miRNA profiles in patients and in IL-10 treated PBMCs**. Representations of miRNAs expressed in HIV-1 patients and IL-10 treated PBMCs. Note the minimal overlap between the two circles.

We next queried how "Patients" changes might resemble primary PBMCs treated with an activating stimulus (anti-CD3; Figure 4) or an inactivating cytokine (IL-10; Figure 5). In PBMCs treated with anti-CD3, 48 miRNAs changes were seen. Amongst these 48 miRNAs, 31 (64%) overlapped with the miRNAs frequently changed in "Patients" (Figure 4). Interestingly, all of the down regulated miRNAs shared between "Infected PBMC" and "anti-CD3" treated PBMCs were also down regulated in the "Patients" (Figure 4). By comparison, IL-10 treated PBMCs showed only 18 miRNA changes (Figure 5), and only a single (6%) miRNA overlapped with the "Patients" (Figure 5). These results suggest that the state of *in vivo *HIV-1 patient PBMCs, as profiled by miRNAs, is more closely modeled by anti-CD3 activation, rather than IL-10 inactivation.

### Several highly abundant T-cell specific miRNAs were down regulated

**Figure 6:**
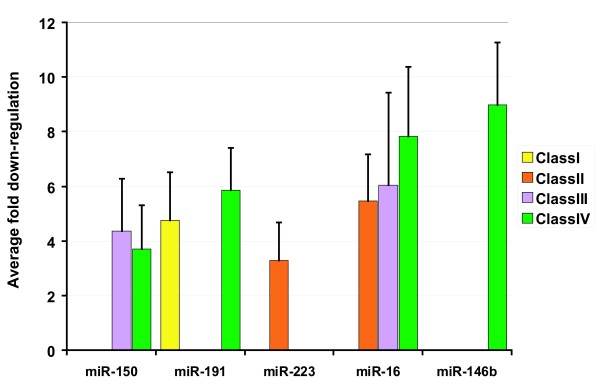
**Down regulation of highly abundant T-cell specific miRNA**. Average fold *in vivo *down-regulation for five highly expressed T-cell miRNAs is graphed.

MiRNA expression is cell-type specific [23]. HIV-1 infection *in vivo *is expected to exert physiologic effects on T-cell function which could be reflected in significant miRNA changes. Elsewhere, miR-223, mR-150, miR-146b, miR-16, and miR-191 have been described to be highly expressed in human T-cells [24,25]. We wondered next whether these abundant T-cell miRNAs could be dysregulated in our patient samples. In our data set, the five T-cell abundant miRNAs showed class specific presentations; and, on average, each was down regulated by 3 to 9 fold (figure 6). Thus *in vivo *HIV-1 infection, in all classes of patients, has sufficient impact to affect significantly the levels of even highly expressed miRNAs. These abundantly expressed T-cell miRNAs are anticipated to provide important biological functions which would be altered accordingly in infected versus uninfected individuals.

## Discussion

We describe here miRNA changes in PBMCs from 36 HIV-1 seropositive individuals categorized into four descriptive classes (Figure 1A). Our findings revealed miRNA signature profiles which are sufficiently distinctive that different classes of HIV-1 infected persons could be distinguished using these biomarkers (Figure 3). Because only a small fraction of PBMCs are infected by HIV-1 *in vivo*, our "Patients" miRNA changes are more likely results of bystander effects [26,27] than outcomes of direct cellular infection by HIV-1. Indeed, the "Patients"-specific miRNA profile did not match well the miRNA changes in virus infected PBMCs (Figure 4).

While the description of signature profiles is interesting, a question remains why do the miRNAs change during HIV-1 infection? The answer is unknown; however, one view is that the virus may benefit from altering the host cell's normal miRNA milieu. This view emerges from the idea that certain host cell miRNAs may serve innate antiviral defenses. Two types of extant findings support the above notion. First, miRNA-processing enzymes such as Drosha and Dicer have been knocked down to reduce the cell's processing of mature miRNAs [22,28,29]. When mammalian miRNAs were thusly reduced, virus replication in cells became more robust. Second, when putative anti-viral miRNAs have been knocked down directly using chemically modified antisense-oligoribonucleotides, or antagomirs [17,19,30], these knock downs also enhanced viral replication. Collectively, these findings are compatible with some cellular miRNAs acting physiologically to suppress viral infection. Indeed, miR-150 and miR-223 have been shown to target the HIV-1 genome, restricting virus expression [17]. Our observed down modulation of these two miRNAs in T-cells (Figure 6) would suggest an *in vivo *setting which favors HIV-1 replication. A second view is that cellular miRNAs could be co-opted by viruses to enhance propagation. Thus, it has been reported that human miR-122 interacts with the 5' UTR of hepatitis C virus (HCV) RNA. MiR-122, rather than antagonizing HCV replication, appears to augment intracellular viral production [31,32]. These two views when taken together argue that down regulation of anti-viral miRNAs and up regulation of virus-augmenting miRNAs may be beneficial objectives for the virus to achieve *in vivo*.

MiRNAs target cellular mRNAs and proteins, and miRNAs are also involved in the differentiation of hematopoietic cells and the regulation of immune cell function and activity [25]. Since one miRNA could potentially target one hundred discrete mRNAs through imperfect complementarity, another outcome of miRNA profile changes may be to alter the landscape of host cell proteins [33]. We note that most "Patients" miRNAs are down regulated (Figure 2), suggesting that the mRNA/protein targets of these miRNAs might be commensurately up regulated *in vivo*. Because many host cell proteins act to modulate HIV-1 replication [16], a careful and detailed analyses of how some of these host factors match as targets of our "Patients" miRNAs would be highly informative.

The above discussions suggest miRNA changes as causative of pathogenic manifestations. On the other hand, it cannot be excluded that the miRNA alterations may simply be consequences of viral pathogenesis. In this respect, HIV/SIV disease progression has been correlated with systemic immune activation [34-37]. We note that our "Patients" miRNA profiles are more consistent with T-cell immune activation (Figure 4) than immune inactivation (Figure 5). Time will tell whether it is miRNA changes that result in immune activation/inactivation or vice versa. We caution that because our PBMC samples have not been fractionated into cellular subsets, some of the differences in miRNA signatures could be explained by in-/out- fluxes of different cell types. Nevertheless, the current picture paints an interplay between cellular miRNAs and viruses which is complex; and one which has evolved into an apparent equilibrium between the host and the pathogen, creating a milieu for moderate and persistent *in vivo *viral infection [38]. Finally, this miRNA analysis, although still in its early stages, might be adapted usefully in the future to staging patients for antiretroviral therapy.

## Materials and methods

### Patients and cells

Normal and human immunodeficiency virus-infected patient PBMCs were obtained from the NIH blood bank and Saint Michael's Medical Center. The study protocol was approved by the St. Michael's Medical Center's Institutional Review Board. Written informed consent was obtained from each subject. The IRB approval letter and the signed, informed consents are available for review. Plasma viral loads were quantified by the Bayer SIV bDNA assay (Bayer Reference Testing Laboratory, Emeryville, CA) [39]. Peripheral blood CD4^+ ^T-cell concentrations were quantified using standard techniques, as previously described [40]. PBMCs were isolated using standard Ficoll separation procedure. Ficoll-purified PBMCs were directly lysed for RNA isolation or stored in liquid nitrogen.

### RNA-primed array-based Klenow extension analysis

RNAs with a cutoff size < 200 nts were hybridized on a microarray printed with 327 probes complementary to mature miRNAs. The probe design and the experimental procedures are the same as previously described [41]. After hybridization, excessive RNA was removed by washing in 0.1 × SSC. Unhybridized probes were removed using exonuclease I (New England Biolabs) for 3 hours. Since the probe design contains a stretch of thymidine, polyadenylation from the 3'end of the hybridized miRNAs was achieved by addition of biotin-label dATP (Enzo Life Sciences). Detection of the labeled miRNA under the 532 nm wavelength was facilitated by addition of streptavidin-conjugated Alexa-flur-555. Data points collected from GenePix 4000B (Molecular Devices) were exported into BRBarray tools (developed by Richard Simon and Amy Peng Lam; ) and JMP software (SAS) for further analysis. Microarray signal normalization was performed using the "median-normalization" procedure. This method is applicable for normalizing arrays in which the majority of data points do not change significantly in values. Essentially, the log-intensities of an array and the reference array are normalized to a median value such that the unchanged gene-by-gene difference between the normalized array and the reference array is 0. The linearity of the microarray readouts has been previously validated using quantitative RT-PCR assays.

## Competing interests

The authors declare that they have no competing interests.

## Authors' contributions

LH, MLY, VdL, and DD carried out the experiments for the studies. LH, MLY, and KTJ drafted the manuscript. KTJ and SMS conceived of the study, and participated in its design and coordination. All authors read and approved the final manuscript.
